# Size Effect of Hydrated Lime on the Mechanical Performance of Asphalt Concrete

**DOI:** 10.3390/ma15103715

**Published:** 2022-05-22

**Authors:** Amjad Albayati, Yu Wang, Jonathan Haynes

**Affiliations:** 1 Department of Civil Engineering, University of Baghdad, Baghdad 10071, Iraq; 2 School of Science, Engineering & Environment, University of Salford, Manchester M5 4WT, UK; y.wang@salford.ac.uk (Y.W.); b.j.haynes@salford.ac.uk (J.H.)

**Keywords:** asphalt concrete, hydrated lime, particle size, moisture susceptibility, resilient modulus, permanent deformation

## Abstract

Despite widespread agreement on the beneficial nature of hydrated lime (HL) addition to asphalt concrete mixes, understanding of the effect of HL particle size is still limited. Previous investigations have focused mainly on two different size comparisons, and so certain guidance for a practical application cannot yet be produced. This study investigates three distinct sizes of HL, in the range of regular, nano, and sub-nano scales, for their effects on the properties of modified asphalt concretes. Five different percentages of HL as a partial replacement of ordinary limestone filler in asphalt concrete mixes were studied for wearing course application purposes. Experimental tests were conducted to evaluate the mechanical properties, including resistance to plastic flow, volumetric properties, moisture susceptibility, resilient modulus, and permanent deformation. The results revealed that a positive correlation exists between the mechanical properties and the fineness of HL particle sizes.

## 1. Introduction

The premature distress of flexible pavements, in the form of permanent deformation, fatigue cracking, and moisture damage, is a persistent issue worldwide, with consequential costs to both road users’ safety and local economies. Using hydrated lime (HL), a reactive mineral filler, to improve aggregate binding and matrix strength has been proven to be an effective solution [1,2]. A life cycle cost analysis [3] showed that HL addition saved approximately 20$ per ton on asphalt concrete, whilst increasing the expected service life of the constructed pavement by 38%. Extensive research has reported the results of the stiffening effect of HL on asphalt mixes [4,5,6] and improvement in fracture-, aging-, and moisture-resistance of asphalt concretes with HL. addition [7,8,9,10,11]. Hydrated lime (also known as calcium hydrate Ca(OH)_2_) reacts with asphalt cement to generate a product of polar components, which help them bond to the surfaces of aggregates and inhibit the hydrophilic polar constituents in the asphalt cement from congregating on the surface of aggregates [12]. In addition, the alkaline nature of HL can neutralize acids to prevent their erosion of the aggregate surface. Moreover, the pozzolanic reactivity of HL can help to remove deleterious agents [13].

Two methods have been adopted to add HL. to asphalt concrete. One is to add it into asphalt cement before mixing with aggregate, while the other is to use it to treat the aggregate before mixing with asphalt cement.

It is well known that the particle size and specific surface area of additives have considerable effects on the surface bonding and interfacial density between the asphalt paste matrix and aggregate. Nano-particle fillers, used as anti-stripping additives, have been investigated for their beneficial effects on asphalt concrete because of their high specific surface area [14,15,16,17,18,19]. A recent study compared the effects of two different types of fillers, CaCO_3_ and HL, at their conventional size and their nano-size in asphalt mixes. It found that much less use of nano-HL filler generated comparable mechanical strength for the mixes than using more normal-sized HL filler. However, in the case of CaCO_3_, the effect of the particle size difference was much less [20]. Nano-silica has also been compared with HL, added to asphalt concrete. It has been found that nano-silica presented up to 10% improvements in tensile strength, fatigue resistance, and moisture susceptibility [14]. Another recent study comparing regular HL (r-HL) and nano-HL (n-HL) of the addition in the range of 5–30% by the weight of bitumen indicated that up to 20% n-HL-modified samples presented steady improvements in tensile strength under both dry and wet conditions [21]. Using n-HL asphalt concrete also showed higher tensile strength under the exposure conditions saturated by de-icing solutions than using normal lime filler [22]. There was also research that studied sub-nano-sized HL effects. A study of warm-mix asphalt concrete, based on the statistical results of the measured tensile strength, showed no significant differences in using sub-nano-HL (sn-HL) or regular HL (r-HL), although positive improvements were observed [23]. Other studies on hot mixed asphalt concrete found that adding 1% of SNHL increased the tensile strength by roughly 10% compared with that using r-HL [24,25]; moreover, 0.5% sn-HL addition obtained a similar anti-stripping capability as that of 1% r-HL addition [24]. All these previous studies provide a qualitative suggestion that (i) HL, due to its active chemical reactivity, demonstrates a better performance than other conventional additives; (ii) the size and specific surface area of mineral additives can interfere with the effects of the nature/type of the additives; and (iii) both the particle size and the nature/type of the additives have disparate effects on asphalt concrete performance-related properties. However, studies of the use of HL can only provide limited qualitative guidance on particle size effects. More informative and implementable quantitative guidance for real-world engineering practice still expects more extensive, specific, and serial experimental data to produce a comprehensive comparison and reliable characterization.

To obtain more information about a wide range of size effects of HL particles on modified asphalt concretes, this study was carried out to directly compare three ranges of HL particle sizes for their effects on the mechanical properties of asphalt concrete, which include resistance to plastic flow, volumetric properties, moisture susceptibility, resilient modulus, and permanent deformation. Five percentages of HL as partial replacements of ordinary limestone filler were investigated, and the asphalt concrete mixes were designed for wearing course applications. A total of 128 specimens with different sizes and geometries were prepared to cover the testing program of this research. Finally, a quantitative characterization model was suggested to predict the HL particle size effect in terms of three size ranges.

## 2. Materials

### 2.1. Raw Materials

The materials used in the study were assessed at first to meet the specifications for the asphalt concrete wearing course application.

#### 2.1.1. Asphalt Cement

The asphalt cement, supplied by Doura refinery in south-west Baghdad, Iraq, was tested based on the requirement of the superpave performance grade. The results shown in Table 1 satisfy the performance grade of PG 64-16.

#### 2.1.2. Aggregate

**Figure 1:**
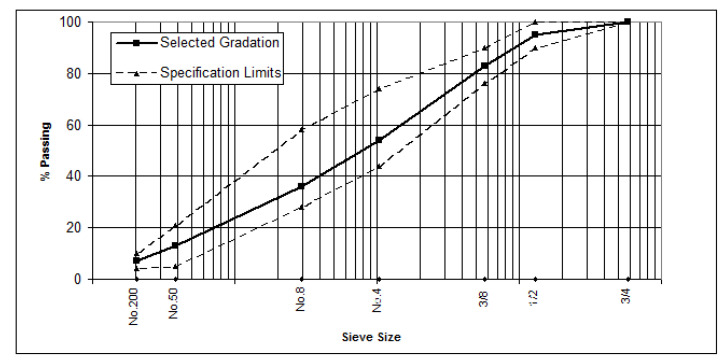
Aggregate gradation.

The aggregate was crushed quartz obtained from the Amanat Baghdad asphalt concrete mix plant located in Taji, north of Baghdad, and originally sourced from the Al-Nibaie quarry. The coarse and fine aggregates were sieved out and recombined for wearing course applications following the specifications of the State Corporation of Roads and Bridges [26]. Figure 1 shows the aggregate gradation curve. Routine tests were performed to evaluate the physical properties of the aggregates, and the results are shown in Table 2.

#### 2.1.3. Filler

A control mix was prepared using limestone dust, which passed sieve No. 200 (0.075 mm) for mineral filler, at a content of 7% by the total aggregate weight. This content was at the midpoint of the range for type IIIA mixes for wearing course applications set by the SCRB specification. Five mixes were prepared using hydrated lime to partially replace the limestone dust using 1.0, 1.5, 2, 2.5, and 3% of the total aggregate weight. The chemical and physical properties of the limestone dust obtained from a local factory are listed in Table 3.

**Figure 2:**
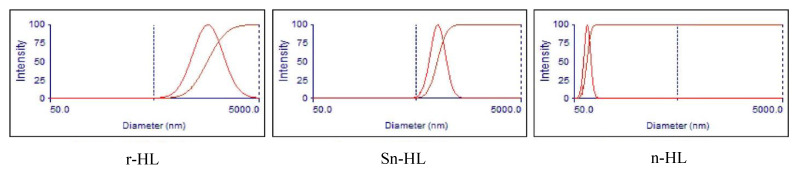
The HL particle size distribution measured by DSL.

Three types of HL were used in this study. A Brookhaven 90 plus instrument was used to apply the Dynamic Light Scattering (DLS) technique to measure HL particle size. Before measuring, HL particles were agitated for 1 min by sonication in deionized water. Figure 2 shows the DLS results for three types of HL, which are regular (r-HL), sub-nano (sn-HL), and nano (n-HL), of average particle sizes 2795 nm, 870 nm, and 93.4 nm, respectively.

**Figure 3:**
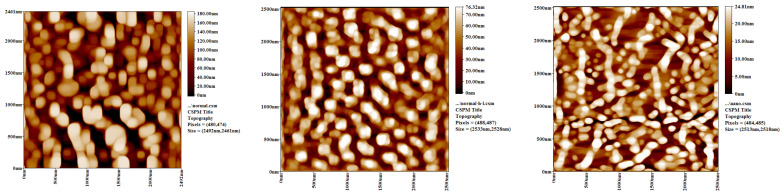
The roughness/topography of three types of HL measured by AFM (**a**) r-HL (**b**) sn-HL, (**c**) n-HL.

All three types of HL were examined using Atomic Force Microscopy (AFM) to assess the surface roughness/topography. The results in Figure 3 show that n-HL had a more uneven surface than did sn-HL and r-HL. The n-HL had the highest surface area to volume ratio (85.5) compared to r-HL (5.41) and sn-HL (26.2). The mean roughness of the r-HL, sn-HL, and n-HL were 43.8 nm, 17.4 nm, and 5.42 nm, respectively.

**Figure 4:**
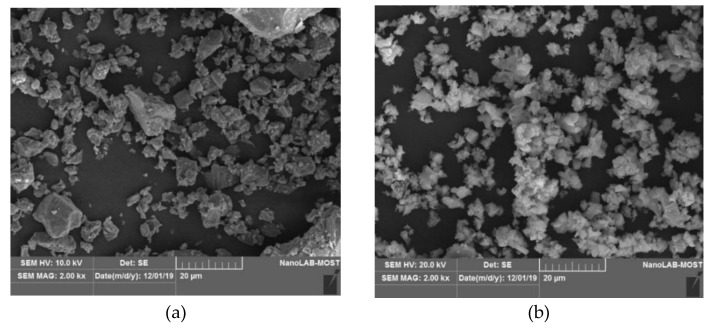

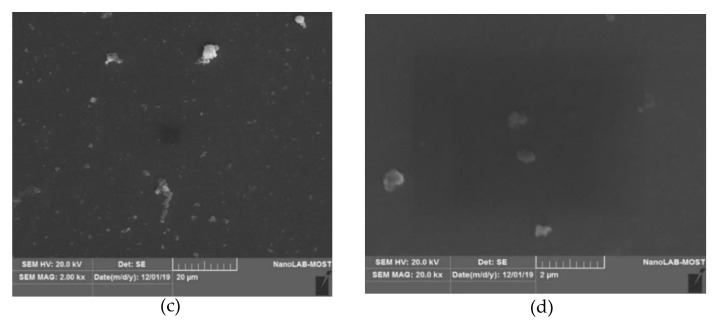
SEM images of the HL particles at 2K×. (**a**) r-HL, (**b**) sn-HL, (**c**) n-HL and (**d**) n-HL at 20K×.

Scanning Electron Microscopy (SEM) provided extra information on the physical configurations and sizes of the HL particles. The SEM images in Figure 4 show the differences in particle size for the three types of HL. The r-HL particles presented an angular shape. The fine particles of the sn-HL tended to agglomerate in the form of a polycrystalline structure. The ultrafine particles of the n-HL examined at two magnification levels presented an irregular shape in a polycrystalline form due to particle agglomeration.

Table 4 lists the chemical composition of the hydrated lime material used in the lime factory to produce the three types of HL based on grain size by grinding into different levels.

### 2.2. HL Addition

The control mix used only limestone dust as the mineral filler, which was 7% of the total weight of the aggregate. Five other mixes were prepared with partial replacement of the lime dust using HL of 1.0, 1.5, 2.0, 2.5, and 3.0% of the total weight of the aggregate. The quantified HL was added to the mixes in the form of a slurry, which was prepared by mixing with 500 mL of water using a shear mixer at 3600 rpm for 10 min. Thereafter, the slurry was poured into the aggregate in a pan and was left for 2 min to blend with and soak the aggregate. The marinated aggregate was then put into an oven at a controlled temperature of 110 °C for 24 h before being used to make asphalt concrete mixes, from which experimental samples were cast. The above-mentioned addition protocol was adopted previously [27,28].

## 3. Experimental Tests

**Figure 5:**
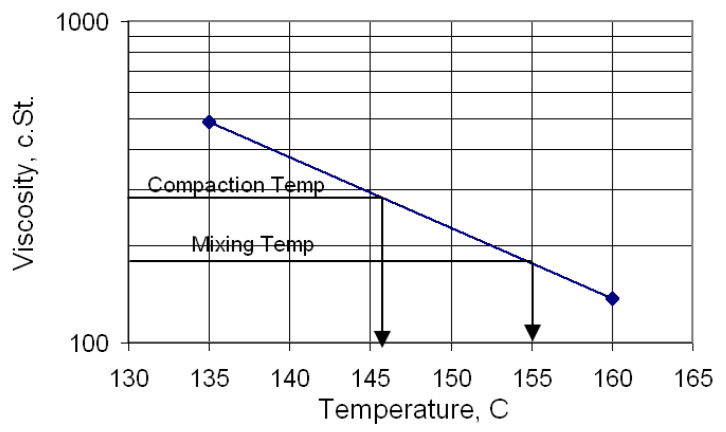
Viscosity–temperature chart of PG 64-16.

Asphalt concrete mixes were prepared with the optimum asphalt content obtained by Marshall properties, stability and flow, air content, density, voids in mineral aggregate (VMA), and voids filled with asphalt (VFA). The optimum asphalt content took a value determined for the r-HL in a previous study (Ahmed, 2013) and was adopted for all the mixes in this study. The aggregate (prepared with or without HL) was first heated in a bowl at 150 °C for 6 h. At the same time, asphalt cement was also heated separately under a controlled temperature of 155 °C for 2 h to achieve a viscosity of 170 c.St according to Figure 5. Thereafter, they were thoroughly mixed for two minutes at 155 °C, and then the mixture was poured into cylindrical molds and transferred into an oven at a controlled temperature of 146 °C, a temperature for compaction, at a viscosity of 280 c.St, for 10 min. The cylindrical molds were of two different sizes. The first was 101.6 mm in diameter and 76.2 mm in height to make the specimens for the Marshall test and indirect splitting tensile test (specimen height 63.5 mm). The second was of the same diameter but 254 mm in height to make the specimens for the resilient modulus and permanent deformation tests (specimen height 203.2 mm).

### 3.1. Marshall Properties

The resistance to plastic flow of the specimens was conducted via a Marshall apparatus following the ASTM (D6927-15) procedure. Marshall stability is the maximum load the specimen can withstand before failure. Marshall flow is the total vertical plastic deformation of the specimen during the test. In addition, the air content and void content in a mineral aggregate can also be obtained from the test according to the bulk specific gravity of specimens (ASTM-D2726-04) and the theoretical specific gravity of the loose mixes (ASTM-D2041-11). Specimens in the cylindrical molds were compacted at each end with 75 blows, which simulated the exposure to high traffic conditions (>10^6^ ESAL). Thereafter, they were de-molded and immersed in water for 30 to 40 minutes at 60 °C before the Marshall tests. Each test was conducted in triplicate.

### 3.2. Indirect Splitting Tensile Strength

The moisture susceptibility of the asphalt concrete mixtures was evaluated following ASTM D 4867-14 for indirect tensile strength (ITS). The specimens of each mix were prepared following the Marshall procedure and compacted at both ends to achieve 7 ± 1% air void content. Six specimens were made for each mix. They were then split into two groups; three as the control were tested directly at 25 °C, while the other three (called conditioned) were subjected to a cycle of freezing and thawing exposure at −18 ± 2 °C for 16 h followed by another 24 h at 60 ± 1 °C before conducting the tensile test at 25 °C. The indirect splitting tensile test applied a compressive load along the diametral axis of the cylindrical specimens at a rate of 50.8 mm/min. The tensile strength (ITS) and tensile strength ratio (TSR) were calculated in terms of Equations (1) and (2).

(1) $$\mathrm{ITS}=\frac{2P}{\pi tD}$$

(2) $$\mathrm{TSR}=\frac{{\mathrm{ITS}}_{c}}{{\mathrm{ITS}}_{d}}$$

where *P* is the ultimate applied load, *t* is the thickness of specimens, *D* is the diameter, and ITS_c_ and ITS_d_ are the tensile strength of the conditioned and control specimens (dry), respectively.

### 3.3. Uniaxial Compressive Deformation and Resilient Modulus

**Figure 6:**
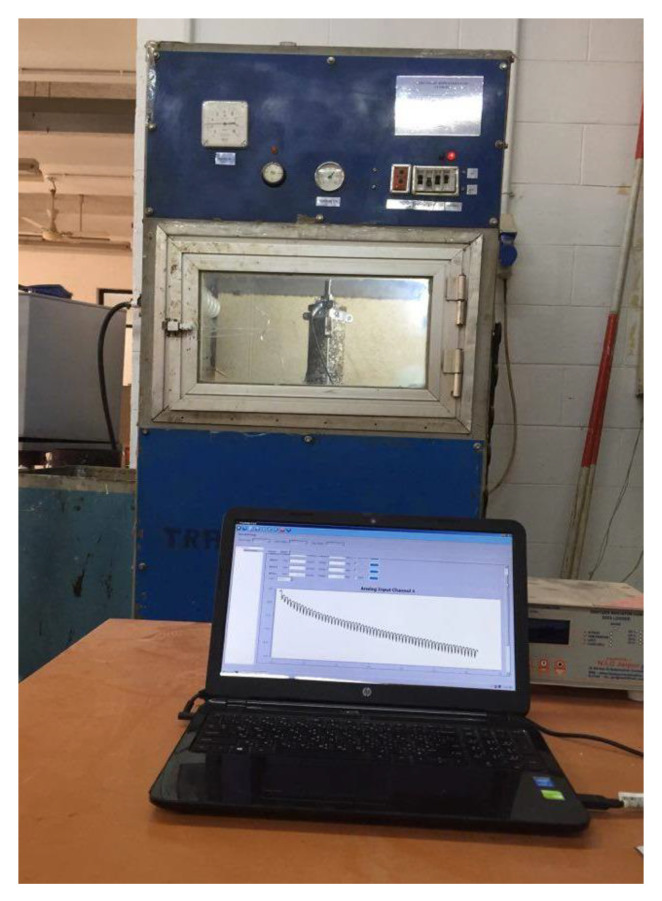
The uniaxial repeated loading test.

Uniaxial compressive tests were conducted using the pneumatic loading system, as shown in Figure 6. A repetitive load was applied in the form of a rectangular wave of a constant frequency of 1 Hz, giving a compressive stress of 0.137 MPa for 0.1 s followed by a rest without load for 0.9 s. Two series of tests were conducted at a controlled temperature of 40 °C to measure permanent deformation and a normal temperature of 20 °C to measure resilient modulus. Full information on specimen preparation for these tests was reported earlier [29]. 

The permanent strain (ε_p_) was calculated according to the recorded permanent axial deformation and Equation (3):

(3) $$\varepsilon_{p}=\frac{p_{d}\times 10^{6}}{h}$$

where *p_d_* is the measured axial permanent deformation, and *h* is the original height of the specimens. In the tests, resilient deflection was measured at the 50–100 cycle repetition. The corresponding resilient strain, *ε_r_*, and resilient modulus, *M_r_*, were calculated using Equations (4) and (5):

(4) $$\varepsilon_{r}=\frac{r_{d}\times 10^{6}}{h}$$

(5) $$M_{r}=\frac{\sigma }{\varepsilon_{r}}$$

where *r_d_* is the axial resilient deflection, *h* is specimen height, and *σ* is axial stress. The measured relationship between the number of load repetitions and the final permanent strain was represented using Equation (6) [30,31], presenting a log-log scale linear trend.

(6) $$\varepsilon_{p}=aN^{b}$$

where *N* is the number of load repetitions, and *a* and *b* are intercept and slope coefficients, respectively.

## 4. Results and Discussion

### 4.1. The HL Size Effect on Marshall Properties

**Figure 7:**
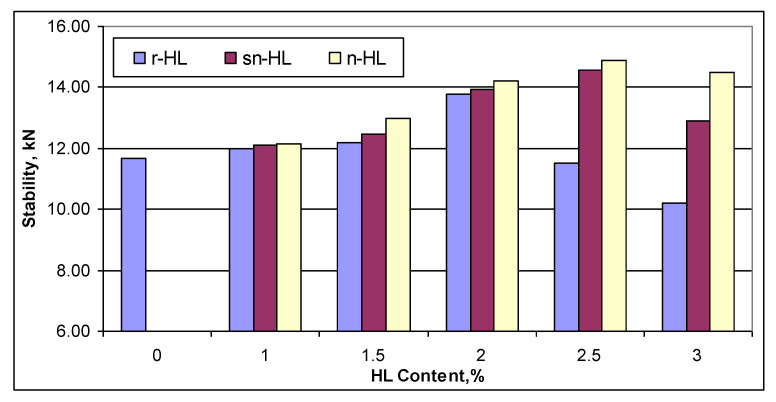
Marshall stability vs. HL type and content.

Detailed results of the measured Marshall properties are shown in Supplementary, Table S1. Figure 7 compares the Marshall stability, which shows that the optimum HL contents were 2% for r-HL but 2.5% each for sn-HL and n-HL. Compared to the control mix, the optimum Marshall stability was improved by 18% using r-HL, 25% using sn-HL, and 28% using n-HL. The improvement rate was higher for the n-HL as compared to sn-HL and r-HL. For each 1 percent increase in HL content, the rate was 1.28 kN for the n-HL, whereas for the sn-HL and r-HL it was 1.15 and 1.06 kN, respectively. The maximum stability was obtained using n-HL due to its high surface area to volume ratio, which not only increased the stiffness of the asphalt binder but also the cohesion force between the aggregate by enhanced infiltration into the aggregate pores.

**Figure 8:**
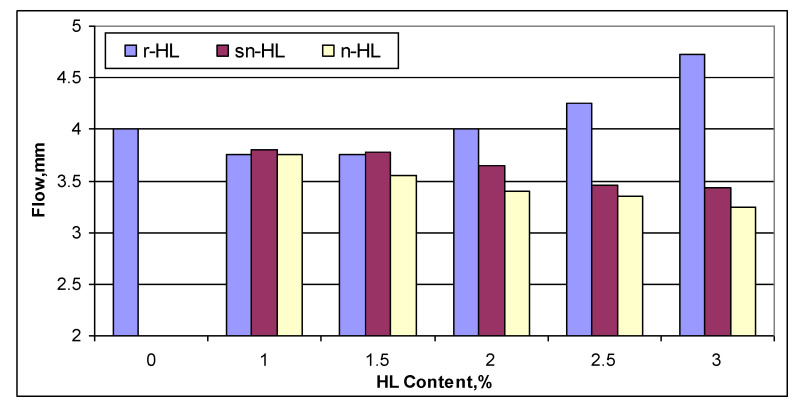
Comparison of Marshall flow.

Figure 8 illustrates the comparison of the Marshall flow results. It can be clearly seen that the finer particle size of the HL, the smaller the Marshall flow measured. The particle size effect had a direct correlation with the HL content. The higher the HL content, the higher the effect of particle size of HL, i.e., the effect of n-HL was highly pronounced at high HL content. The opposite effect of the n-HL and sn-HL content in the Marshall flow as compared to the r-HL could be attributed to the superior ability of the ultrafine and fine particles of the n-HL and sn-HL, respectively, to stiffen the asphalt concrete mixes and hence to decrease the flow values, but within the specification limits of the Marshall flow values (2–4 m).

**Figure 9:**
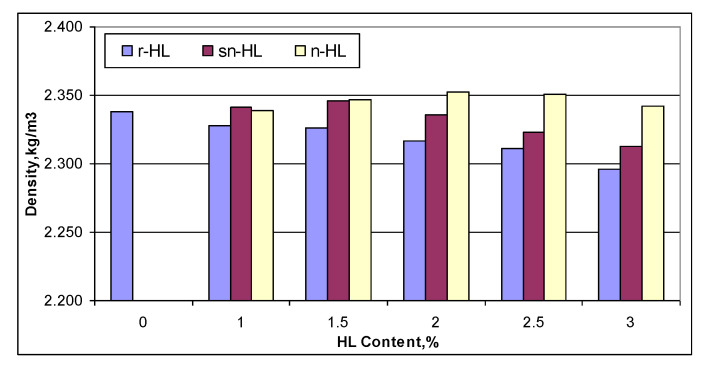
Comparison of density.

Figure 9 illustrates the comparison of density variation. The n-HL had the highest density when HL content started from 1.5%, and its maximum was at the HL of 2%. A general trend is that the finer the HL particles, the higher the density and higher the HL content for mixes at their respective maximum density.

**Figure 10:**
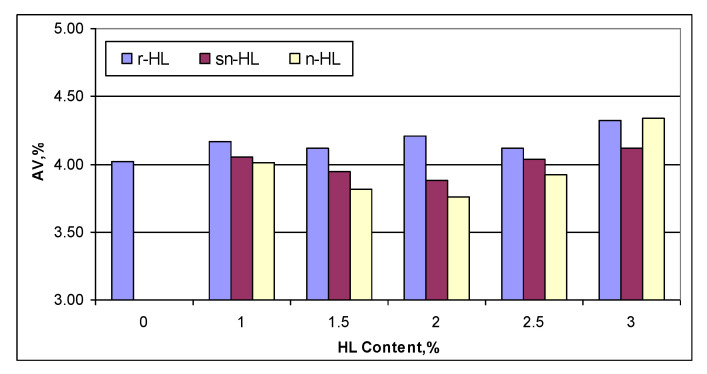
Comparison of air voids.

Figure 10 compares the air void content. Both sn-HL and n-HL had their respective least air content at 2% HL content. In general, the finer the HL particles, the lower the air content, up to 2.5%. The results highlight a high level of compaction required by the mixes of high HL content.

**Figure 11:**
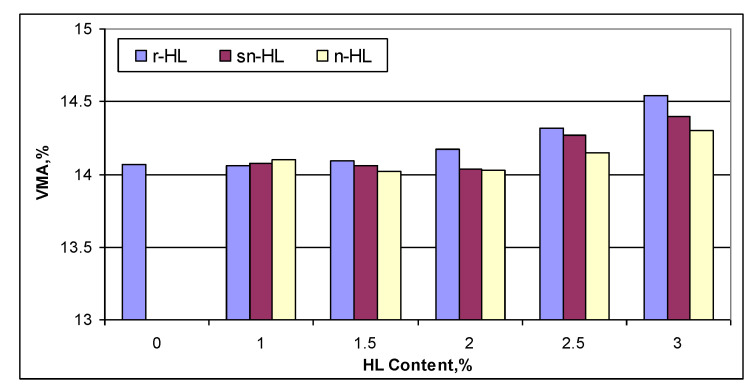
Comparison of VMA.

Figure 11 compares the VMA values for different types and contents of HL It shows that n-HL had the lowest VMA when HL content started from 1.5%. A high HL content, starting from 2.5%, generated a high VMA, which reflects the need for a high level of compaction in pavement applications.

**Figure 12:**
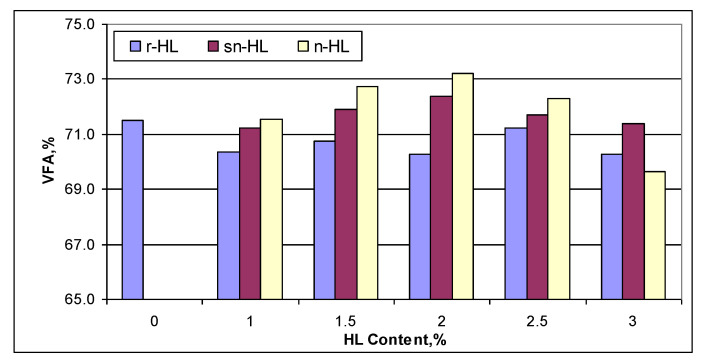
Comparison of VFA.

Figure 12 compares the percentage of the filled voids in the mineral aggregate by asphalt binder (VFA). It shows that when the content of n-HL and sn-HL was more than 2%, VFA started to decrease, which indicated that high HL content reduced the efficiency of the penetration into the micropores of aggregate because of the increased stiffness of the binder. For r-HL, the VFA was generally lower than the control mix. The highest VFA was at 2% of n-HL or sn-HL, whereas for the r-HL, it was at 2.5%.

In general, using small HL particles will enhance the Marshall properties of asphalt concrete. The results of this study suggest that the optimum usage of the sn-HL and n-HL is in the range of 2~2.5% in terms of the three key benefits on Marshall stability, Marshall flow, and volumetric properties. The matter which reflects the efficient role of the fine HL types (n-HL and sn-HL) is that they play an important role in extending the stiffness of the mastic (combination of asphalt cement and filler) and effectively filling the voids within stone skeleton of the mixture, resulting in high Marshall stability. Although a high replacement rate of HL may require a high compaction effort (as the VMA value is high), it was lower, however, in the case of n-HL and sn-HL in comparison with r-HL due to the tiny nature of n-HL and sn-HL.

### 4.2. The HL Size Effect on Tensile Strength and Moisture Susceptibility

**Figure 13:**
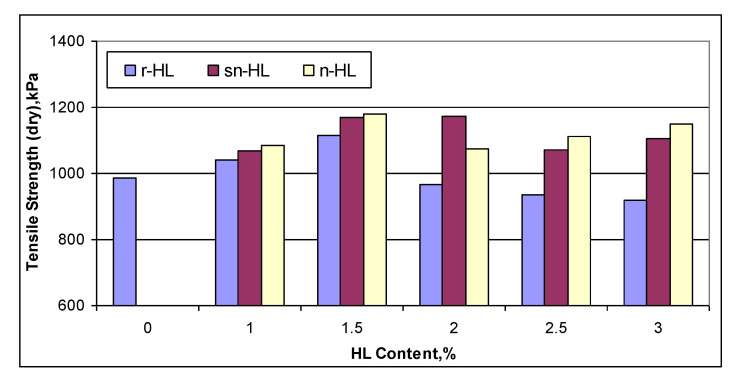
The dry tensile strength of the samples in the moisture susceptibility tests.

Figure 13 compares the tensile strength of the control samples without moisture exposure (dry). The measurement data are listed in Supplementary Table S2. Figure 13 shows that the n-HL mix had the highest tensile strength at 1.5% HL content. Compared to the specimens using no HL, the r-HL, sn-HL, and n-HL specimens showed an improvement in the average value of splitting tensile strength by 14.6, 30.4, and 31.6 percent, respectively.

**Figure 14:**
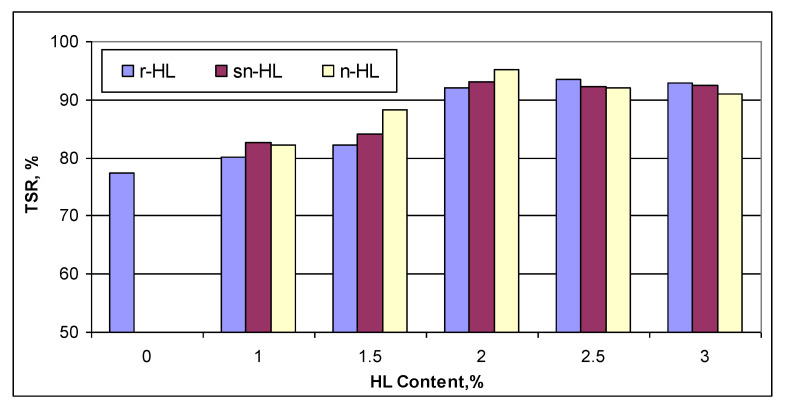
The indirect tensile strength ratio.

Figure 14 compares the calculated TSR It can be seen that the use of HL improved the moisture susceptibility in general, and a positive correlation with particle size could be observed until 2% HL content for sn-HL and n-HL, and 2.5% for r-HL. As the minimum acceptable TSR is 80%, the use of HL satisfied the specification requirement. The maximum gain in TSR was 18.5% at 2% n-HL in comparison with the control mix (no hydrated lime). The improvement in moisture susceptibility was because HL particles could be efficiently combined with the viscous components exposed to oxidative aging and thus help to reduce the process under moisture exposure. Conversely, HL particles are involved in the cation exchange in agglomeration and pozzolanic reactions in asphalt mixtures; their products have a less hydrophilic nature, which also helps reduce the moisture susceptibility of asphalt concrete [32,33,34]. The current study confirmed that a small particle size will enhance this capability but only for certain proportions. In particular, the high ratio of surface area to volume of n-HL particles promoted the degree of cation exchange reactions and consequently the hydrophobic benefit.

### 4.3. The HL Size Effect on Resilient Modulus (Mr)

**Figure 15:**
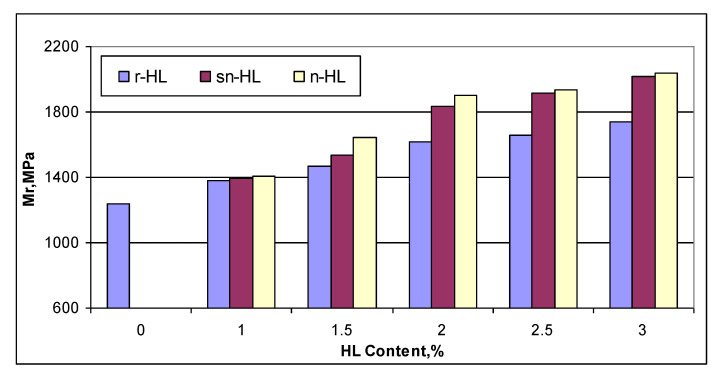
Comparison of the resilient modulus.

Figure 15 compares the data in Supplementary Table S3. It can be seen that a direct correlation existed between the resilient modulus, proportion of HL addition, and particle size. In general, Mr increased with HL addition and a decrease of the HL particle size. Compared to the control mix without HL, the use of n-HL, sn-HL, and r-HL resulted in an increase of Mr by approximately 20%, 17%, and 9%, respectively, per 0.5% addition, up to 2%. Reduced improvements continues to occur up to 3% HL content. This result agrees with the basic mechanisms of materials and asphalt rheology. Under axial compressive loading, tensile stress existed in the transverse direction at the mid height of the specimens. As aggregate particles were unable to carry the tensile force, the strength of the asphalt concrete primarily depended upon the cohesion of the asphalt cement. The ultra-fine HL particles increased the cohesion and stiffness of the asphalt matrix and resulted in the effective increase of the resilient modulus.

### 4.4. The HL Size Effect on Permanent Deformation

**Figure 16:**
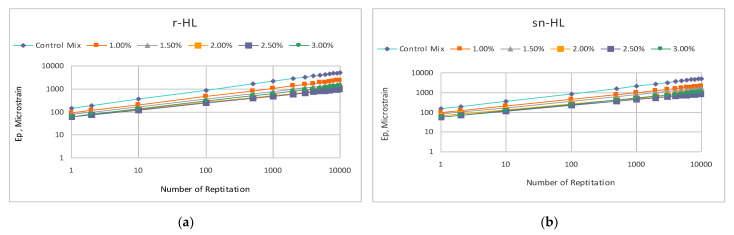

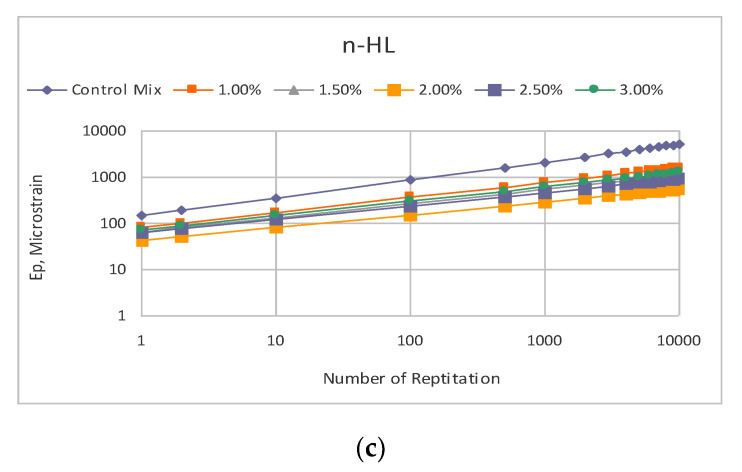
Comparison of the permanent deformation. (**a**) r-Hl, (**b**) sn-HL and (**c**) n-HL.

**Figure 17:**
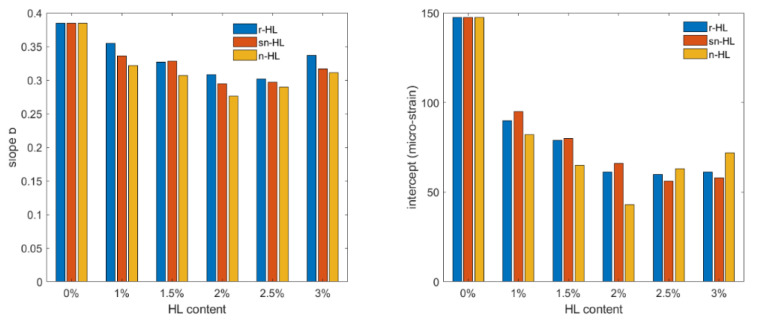
Comparison of the permanent deformation parameters, intercept and slope.

Figure 16 shows the test results of the permanent deformation measurement under uniaxial repetitive load (plotted on a log–log scale). Using Equation (6) to represent the relationship, two parameters, i.e., intercept and slope, are related to the permanent deformation potential of the material. Figure 17 compares the slope and intercept parameters of the fitting lines. The results indicate that HL addition in general improved the rutting resistance of asphalt concrete. Using nano-sized particles produced the highest improvement, and at an HL content of 2%, the lowest values of the slope and intercept at 2% n-HL were much lower than the optimum values of the r-HL at 2.5%. It can be shown that at 2% NHL, the permanent strain at 10,000 load repetitions was about 546 micro-strain, which was 43.7% and 36.7% lower than that of the r-HL and sn-HL at their optimum content of 2.5% (Table 5). Overall, compared to the control mix without HL addition, the average improvement on the permanent strain at 10,000 load repetitions was approximately 71.3, 73.5, and 78.9 micro-strain, respectively, for the use of r-HL, sn-HL, and n-HL. The above results revealed the superior role of the finest HL type in producing a high stiffness asphalt concrete and increasing stone–stone contact, which resulted in efficient distribution as well as reduction in the stresses and strains in the pavement structure created by traffic loading, consequently reducing the possibility of a high temperature rutting mode of failure.

**Figure 18:**
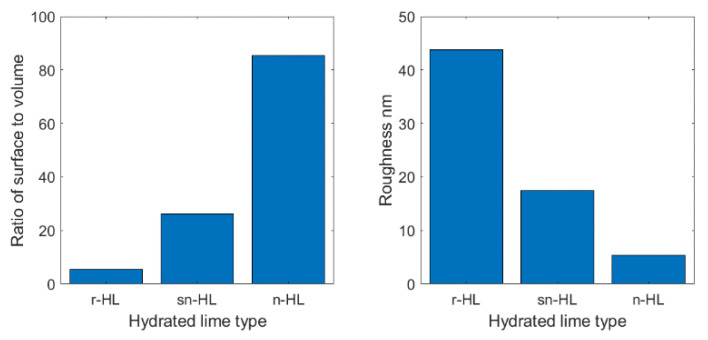
The physical properties of HL particles.

**Figure 19:**
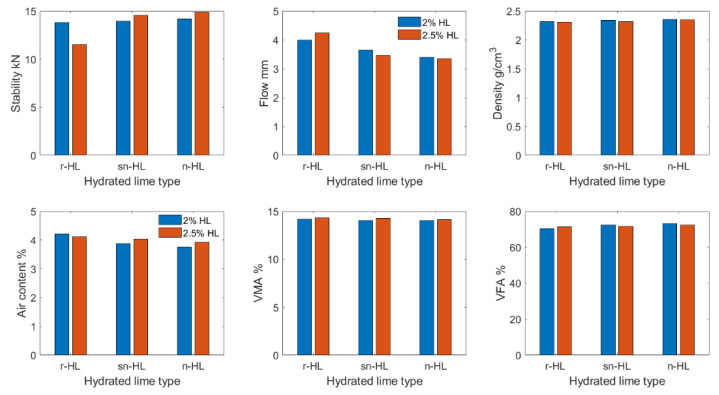
Marshall Properties at 2.0 and 2.5% HL.

**Figure 20:**
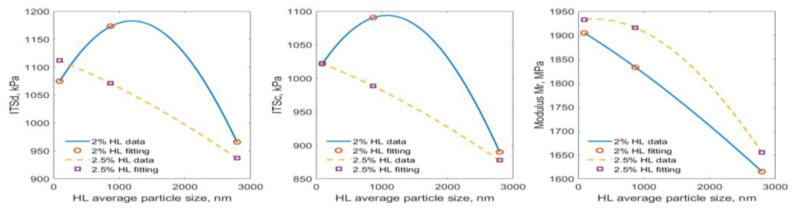
Gaussian characterization of tensile strength and resilient modulus at 2.0 and 2.5% HL.

To summarize the experimental tests, and to assist practitioners in making decisions about deploying hydrated lime in asphaltic concrete, Figure 18 compares the physical properties of the HL particles, and Figure 19 and Figure 20 compare the measured properties at 2.0 and 2.5% addition of the three types of HL The Gaussian formula (Equation (7)) is suggested to quantify the HL particle size effect on the two key mechanical properties in Figure 20. Table 6 lists the three fitting parameter values.

(7) $$y=ae^{-{\left(\frac{x-b\;}{c}\right)}^{2}}$$

## 5. Conclusions

From the experimental study, the following conclusions can be drawn:

1. The surface to volume ratio of the hydrated lime particles increases from regular size to the nano level, and, conversely, their roughness decreases.
2. A positive correlation exists between several asphalt concrete mechanical properties and the fineness of hydrated lime particles.
3. Using sub-nano-hydrated lime and nano-hydrated lime generates an optimum improvement on the Marshall properties and volumetric properties of the modified asphalt concrete at a content in 2~2.5%.
4. The resilient modulus increases with HL addition, and decrease of the HL particle size. Up to 2% HL, the improvement rate are +250, +218.5 and +118 mPa for each 0.5 percent addition of n-HL, sn-HL and r-HL, respectively.
5. When subjected to a repetitive loading, nano-hydrated lime asphalt concrete demonstrates the best rutting resistance performance with an optimum content at 2%. The sub-nano-hydrated lime mix shows a much higher deformation starting from initial load up to 10,000 repetitions, and its optimum content is 2.5%.
6. The sub-nano-hydrated lime mix shows the best improvement in moisture susceptibility at 2% content, but this is marginal compared to nano-hydrated lime concrete. So overall, 2~2.5% replacement of the conventional mineral filler using nano-hydrated lime is proposed to be the optimum mix for the surface wearing course, from this study.
7. A Gaussian characterization model well represents the HL size effect on asphalt concrete major mechanical properties. The model can be used in practice for pavement material and structural design for to optimize the targeted applications and cost.

## 6. Recommendations for Future Research

Based on the findings of this study, further studies are recommended to consider the following:

1. The content and size effect of HL on the fatigue mode of failure for asphalt concrete;
2. Life cycle cost analysis of the hot mix asphalt wearing course considering the content and size effect of HL.

## Figures and Tables

**Table 1:** Physical properties of the asphalt cement.

Binder Condition	Parameters	Test Temperature °C	Measurement Data	Specification (AASHTO M320-05)
Original	Flash Point, °C	-	298	230, min
	Viscosity, Pa.s	135-	0.487	3, max
	DSR, G/sinδ at 10 rad/s, kPa	58	3.3522	1.00, min
		64	2.020	
		70	0.889 *	
RTFO Aged	Mass Loss, %	-	0.654	1%, max
	DSR, G/sinδ at 10 rad/s, kPa	58	4.1596	2.2, min
		64	3.1483	
		70	1.9809 *	
PAV Aged	DSR, G.sinδ at 10 rad/s, kPa	28	4684	5000, max
		25	6477 *	
	BBR, Creep Stiffness, mPa	−6	134.0	300, max

* failed to satisfy the specification requirement.

**Table 2:** Physical properties of aggregates.

Property	ASTM Design	Test Results	SCRB Specification
Coarse aggregate			
Bulk specific gravity	C-127	2.646	-
Apparent specific gravity		2.656	-
Water absorption (%)	-	0.14	-
Percent wear by Los Angeles abrasion (%)	C-131	19.7	30 max
Soundness loss by sodium sulfate solution (%)	C-88	3.4	12 max
Flat and elongated (5:1) (%)	D4791	4	10 max
Fractured pieces (%)	D5821	97	90 min
Fine aggregate			
Bulk specific gravity	C-128	2.561	-
Apparent specific gravity		2.612	-
Water absorption (%)		0.782	-
Sand equivalent (%)	D2419	55	45 min
Clay lump and friable particles (%)	C-142	1.3	3 max.

**Table 3:** Properties of the limestone dust used for mineral filler.

Chemical Composition, %						
CaO	SiO_2_	Al_2_O_3_	MgO	Fe_2_O_3_	SO_3_	L.O.I
29	10	6	16	1	0.12	37
**Physical Properties**						
Specific Gravity		Surface Area * (m^2^/kg)		Passing Sieve No. 200 (0.075) %		
2.84		247		95		

* Blain air permeability method (ASTM C204).

**Table 4:** Chemical properties of hydrated lime.

Chemical Composition, %						
CaO	SiO_2_	Al_2_O_3_	MgO	Fe_2_O_3_	SO_3_	L.O.I
69.5	1.0	-	2.0	-	150	26

**Table 5:** Permanent micro-strain (ε_p_) at 10,000 load repetitions.

		HL Content, %					
		0	1.0	1.5	2.0	2.5	3.0
HL type	r-HL	5130	2364	1617	1051	970	1364
	sn-HL		2098	1585	1183	863	1075
	n-HL		1592	1099	546	911	1263

**Table 6:** The fitting data of the Gaussian formula.

Mix	Property	a	b	c
2% HL	ITS_d_	1183	1195	3552
	ITS_c_	1094	1081	3773
	Mr	2073	−3664	1.293 × 10^4^
2.5% HL	ITS_d_	1174	−2518	1.118 × 10^4^
	ITS_c_	1077	−2665	1.206 × 10^4^
	Mr	1934	244.2	6462

## Data Availability

The raw data required to reproduce these findings are available in the Supplementary Materials.

## Supplementary Materials

The following supporting information can be downloaded at: https://www.mdpi.com/article/10.3390/ma15103715/s1, Table S1: Marshall property measurement; Table S2: The indirect tensile strength with and without moisture exposure; Table S3: Results of the resilient modulus (Mr) tests.
