# Supplementary material for: Size Effect of Hydrated Lime on the Mechanical Performance of Asphalt Concrete
Source: Materials (Basel). 2022 May 22;15(10):3715. doi: 10.3390/ma15103715 (PMC9146764; doi:10.3390/ma15103715)
Supplement: Supplementary file 1 [file materials-15-03715-s001.zip › materials-1732465-supplementary.pdf]

materials

MDPI

Supplementary Materials

Size Effect of Hydrated Lime on the Mechanical Performance of Asphalt Concrete

Amjad Albayati [1,*], Yu Wang [2] and Jonathan Haynes [2]

[1] Department of Civil Engineering, University of Baghdad, Iraq
[2] School of Science, Engineering & Environment, University of Salford, UK
* Correspondence: Corresponding author: a.khalil@uobaghdad.edu.iq

Table S1. Marshall property measurement.

VFA %	VMA %	Air Voids %	Density gm/cm^3	Flow mm	Stability kN	OAC %	HL type	HL Content %
71.50	14.07	4.01	2.338	4.0	11.67	4.9	Control	0
70.34	14.06	4.17	2.328	3.75	12.0	4.9	r-HL	1
71.24	14.08	4.05	2.341	3.80	12.10		SNHL	
71.56	14.10	4.01	2.339	3.75	12.15		NHL	
70.76	14.09	4.12	2.326	3.75	12.2	4.9	R-HL	1.5
71.91	14.06	3.95	2.346	3.78	12.45		SNHL	
72.75	14.02	3.82	2.347	3.55	12.98		NHL	
70.29	14.17	4.21	2.317	4.0	13.79	5.0	r-HL	2.0
72.36	14.04	3.88	2.336	3.65	13.95		SNHL	
73.20	14.03	3.76	2.352	3.40	14.20		NHL	
71.23	14.32	4.12	2.311	4.25	11.51	5.2	r-HL	2.5
71.69	14.27	4.04	2.320	3.46	14.56		SNHL	
72.30	14.15	3.92	2.350	3.35	14.87		NHL	
70.29	14.54	4.32	2.296	4.73	10.22	5.2	r-HL	3.0
71.39	14.40	4.12	2.310	3.44	12.92		SNHL	
69.65	14.30	4.34	2.340	3.25	14.48		NHL	

Table S2. The indirect tensile strength with and without moisture exposure.

Gains %	TSR %	ITS_c kPa	ITS_d kPa	HL Type	HL Content %
---	77.5	763.2	984.6	Control	0%
3.2	80.1	835.0	1041.8	r-HL	1%
6.3	82.7	883.2	1068.7	SNHL	
5.7	82.2	890.8	1083.9	NHL	
5.7	82.2	917.0	1114.9	r-HL	1.5%
7.8	84.1	981.8	1168.0	SNHL	
12.2	88.3	1042.5	1181.1	NHL	
15.9	92.1	890.1	966.0	r-HL	2.0%
16.8	93.2	1090.8	1173.5	SNHL	
18.5	95.1	1021.8	1074.9	NHL	
17.2	93.6	877.7	937.0	r-HL	2.5%
16.0	92.3	988.7	1071.4	SNHL	
15.8	92	1022.5	1112.1	NHL	
16.6	92.9	853.6	918.4	r-HL	3.0%
15.2	92.5	1029.4	1105.9	SNHL	
14.9	91.1	1046.6	1149.4	NHL	

Table S3. Results of the resilient modulus (Mr) tests.

Mr<br>MPa	HL type	HL Content<br>%
1235	Control	0%
1379	r-HL	1%
1396	SNHL	
1405	NHL	
1471	r-HL	1.5%
1536	SNHL	
1641	NHL	
1615	r-HL	2.0%
1833	SNHL	
1905	NHL	
1655	r-HL	2.5%
1916	SNHL	
1933	NHL	
1742	r-HL	3.0%
2016	SNHL	
2039	NHL	
